# Research on the Improved ICP Algorithm for LiDAR Point Cloud Registration

**DOI:** 10.3390/s25154748

**Published:** 2025-08-01

**Authors:** Honglei Yuan, Guangyun Li, Li Wang, Xiangfei Li

**Affiliations:** 1 Institute of Geospatial Information, Information Engineering University, Zhengzhou 450001, China; honglei220@163.com (H.Y.); wangli_chxy@163.com (L.W.); 2 Shanxi Institute of Surveying Mapping and Geoinformation, Taiyuan 030001, China; 15034127513@163.com

**Keywords:** laser scanners, point cloud, scanning incidence angle, weighting function, weighted ICP registration

## Abstract

Over three decades of research has been undertaken on point cloud registration algorithms, resulting in mature theoretical frameworks and methodologies. However, among the numerous registration techniques used, the impact of point cloud scanning quality on registration outcomes has rarely been addressed. In most engineering and industrial measurement applications, the accuracy and density of LiDAR point clouds are highly dependent on laser scanners, leading to significant variability that critically affects registration quality. Key factors influencing point cloud accuracy include scanning distance, incidence angle, and the surface characteristics of the target. Notably, in short-range scanning scenarios, incidence angle emerges as the dominant error source. Building on this insight, this study systematically investigates the relationship between scanning incidence angles and point cloud quality. We propose an incident-angle-dependent weighting function for point cloud observations, and further develop an improved weighted Iterative Closest Point (ICP) registration algorithm. Experimental results demonstrate that the proposed method achieves approximately 30% higher registration accuracy compared to traditional ICP algorithms and a 10% improvement over Faro SCENE’s proprietary solution.

## 1. Introduction

Since the 1990s, point cloud registration technology has undergone a leapfrog development from manual intervention to fully automatic algorithms, forming a complete technical system ranging from traditional geometric algorithms to deep learning integration. Its core goal is to achieve a precise alignment of multi-view point cloud data through spatial transformation matrices [1,2,3]. Traditional methods revolve around the ICP algorithm [4,5], which was proposed by Besl and McKay in 1992. Due to its mathematical simplicity, it became the gold standard for fine registration. However, its heavy reliance on initial poses often leads to local convergence issues, and its computational complexity grows exponentially with the scale of point clouds [6]. To overcome these limitations, researchers have successively proposed various improvements, gradually forming a classic two-stage paradigm of coarse registration and fine registration, each demonstrating unique advantages and limitations in different application scenarios. Coarse registration primarily addresses the ICP’s dependency on initial poses in registration [7,8], and can be broadly categorized into feature-matching-based methods, exhaustive-search-based methods, and probability-distribution-based methods. Feature-matching-based methods, represented by Sample Consensus Initial Alignment (SAC-IA) [9], extract point cloud features (e.g., FPFH [10]) using a random sampling consensus strategy, establish correspondences, and compute initial transformation matrices. While these methods excel in structured scenes, they are sensitive to noise and unstructured environments, and tend to fail in low-overlap regions due to their reliance on the discriminability of feature descriptors. Exhaustive-search-based methods, typified by Four-Points Congruent Sets (4PCS) [11,12], leverage the geometric invariants of rigid transformations (e.g., segment ratios, distance consistency, etc.) to search for coplanar four-point sets in source and target point clouds, generating candidate transformation matrices through congruence constraints. Their strength lies in robustness to low-overlap point clouds, but their computational complexity increases exponentially with the point cloud scale. Probability-distribution-based methods, exemplified by Normal Distributions Transform (NDT) [13], partition point clouds into voxel grids and compute normal distribution probability density functions for each grid, optimizing transformation parameters by maximizing the likelihood function. Their feature-free matching characteristic creates advantages in dynamic environments, but grid size selection significantly impacts accuracy [14,15]. Fine registration methods primarily enhance registration accuracy and precision through algorithmic optimizations based on ICP. The ICP algorithm achieves point cloud registration by iteratively searching for nearest neighbor point pairs and optimizing rigid transformation matrices [16]. Its core steps include nearest neighbor search, transformation matrix solving, and iterative convergence. However, ICP’s heavy reliance on initial poses makes it prone to local optima. Researchers have proposed improved algorithms such as GICP, Go-ICP, and NICP. GICP enhances robustness to point cloud noise by introducing covariance matrices [17]. Go-ICP employs a branch-and-bound strategy to achieve global optimality in point cloud searches [18]. Normal ICP (NICP) improves matching accuracy for curved surfaces by integrating normal vector information [19].

In recent years, deep learning technologies have broken traditional paradigms. Algorithms such as PointNetLK [20] achieve real-time registration at the millisecond level through end-to-end feature learning. Zhou Ruqin’s team systematically reviewed advancements in deep learning registration strategies, including pose regression and correspondence resolution, and highlighted the potential of transformer models in cross-scene generalization [21]. However, the generalization capabilities of these models remain constrained by the completeness of training data.

In both traditional and deep learning-based registration methods, the core tasks revolve around objective function optimization. However, existing registration algorithm theories often assume that input point cloud quality is uniform and consistent, applying equally weighted treatment to all input data during objective function optimization. This assumption neglects potential significant variations in point cloud quality under different scanning conditions, resulting in limited further improvements in registration accuracy in practical applications. As research on point cloud registration technology deepens, researchers have gradually recognized that point cloud scanning quality remains one of the key variables affecting registration accuracy.

The quality of point clouds acquired using laser scanners is influenced by multiple factors. First, the material properties of object surfaces significantly affect scanning results [22]. For example, differences in reflectivity between materials can impact ranging accuracy and introduce noise points. However, in many practical scanning scenarios, accurately quantifying material-induced quality variations is challenging, especially in small-scale scans, where targets are often homogeneous or similar in material type. Thus, this study temporarily excludes material-related effects. Scanning distance is another critical factor [23,24]. Research shows that, as the scanning distance increases, ranging accuracy degrades sharply, leading to reduced point cloud quality. Additionally, scanning incidence angle [24,25,26] (the angle between the laser beam and the surface normal) plays a significant role. Studies by Zámečníková and Al-Saedi et al. demonstrate that larger incidence angles correlate with higher noise levels and poorer point cloud quality. This study focuses on small-scale industrial point cloud registration, with scanning ranges typically under 20 m. Under such conditions, variations caused by scanning distance or material heterogeneity are negligible due to the limited scale and material uniformity of the scanned objects. Therefore, this study prioritizes analyzing the impact of laser incidence angles on registration accuracy and proposes an improved ICP algorithm weighted by incidence angles.

The remainder of this article is structured as follows: Section 2 describes the impact of incidence angle on point cloud accuracy and quantifies the degree of influence through specific experiments. Section 3 discusses different weighting methods for point clouds with varying incidence angles and the corresponding weight assignment approaches for point pairs during registration, along with the principles of the weighted ICP algorithm and its data processing workflow. Section 4 presents an experimental analysis, detailing the testing procedures and accuracy evaluation metrics. Section 5 provides conclusions and future prospects, summarizing the study and discussing potential research directions for point cloud registration technologies.

## 2. Impact of Incidence Angle on Point Cloud Accuracy

**Figure 1:**
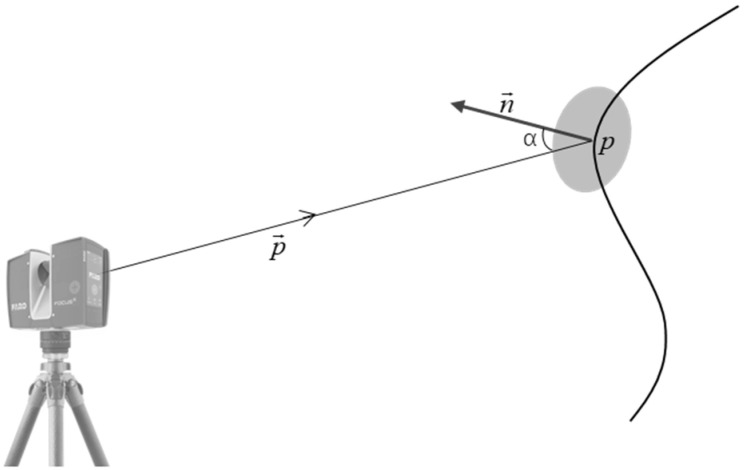
Schematic diagram of incidence angle in point cloud data.

Before analyzing the impact of laser scanning incidence angles on point cloud accuracy, it is essential to clarify the definition and computational methodology of point cloud incidence angles. The incidence angle of a point cloud refers to the angle between the laser beam emitted by the scanner and the normal vector of the point cloud (as illustrated in Figure 1). Specifically, for a given point p in the point cloud, its incidence angle α is derived from the angle between vectors $\vec{n}$ and $\vec{p}$, as defined by Equation (1) ($0\leq \alpha <90^{{}^{\circ}}$). The normal vector $\vec{n}$ at point p is determined by fitting a local surface using neighboring points around p. The laser beam direction is represented by the vector $\vec{p}$ from the scanner’s center coordinates to point p.

(1) $$\alpha =\arccos(\frac{-\vec{p}\cdot \vec{n}}{\left|\vec{n}\right|\left|\vec{p}\right|})$$

Currently, there is no unified evaluation standard for assessing the impact of different incidence angles on point cloud accuracy. In existing research, some researchers have evaluated point cloud precision by using ranging error analysis [26], while others have used the denoising rate of point clouds at various incidence angles as an evaluation metric [24]. However, these methods may have certain limitations in practical applications and cannot fully reflect the influence of incidence angles on point cloud accuracy. To address this issue, this study uses a novel evaluation approach that analyzes the RMS of fitting results for the same reference plane under different incidence angles, thereby quantitatively assessing the variations in point cloud accuracy caused by incidence angle differences. During the experiments, to ensure the comparability of test results, we employed the same model of laser scanner (Faro Focus 350 terrestrial laser scanner with a ranging error of ±1 mm) and a precision-machined reference plane (a steel plate with a flatness better than 0.01 mm). This setup effectively eliminates additional errors introduced by equipment variations or inconsistencies in reference planes, thereby ensuring the accuracy and reliability of the experimental results.

**Figure 2:**
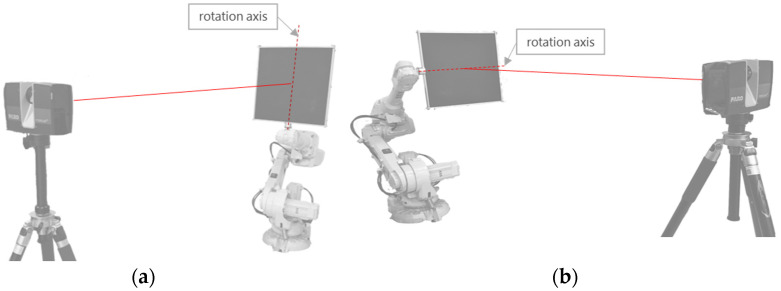
Reference plate scanning method: (**a**) horizontal rotation experiment, and (**b**) vertical rotation experiment.

The experiment was conducted using both horizontal and vertical rotation testing methods, as illustrated in Figure 2. The procedure involved the following steps: First, the precision-machined reference plate was securely mounted onto an industrial robotic arm using specialized fixtures. Subsequently, the Faro Focus 350 laser scanner was positioned approximately 3 m away from the reference plane. The scanner’s height was carefully adjusted to ensure that its optical center was aligned horizontally with the center of the reference plate.

For the horizontal rotation experiment, the procedure was conducted as follows: First, the industrial robotic arm was rotated to a position of the reference plane that was approximately perpendicular to the laser scanner’s incident beam, with this position marked as the 0° incidence angle reference point, followed by the initial scan measurement. Subsequently, the robotic arm was controlled to rotate 10° counterclockwise about the vertical axis of the reference plane, with this new position marked as the 10° incidence angle position, and the scanner was activated again for measurement. Following the same operational sequence, a scan was performed at each incremental rotation until reaching the 80° position, thereby completing the horizontal scanning experiment. For the vertical rotation experiment, a similar methodology was employed: the robotic arm was rotated about the horizontal axis of the reference plane, with scans performed at various angles. Measurements were similarly recorded at 10° intervals from 0° to 80° to obtain a comprehensive dataset.

Using the two aforementioned rotation methods, we ultimately obtained 18 complete sets of scanning data. Each dataset underwent the following preprocessing steps: First, the raw point cloud was segmented into two parts: the local point cloud corresponding to the reference plate area, and the remaining background data. Only the reference plate-related data were retained to minimize any unnecessary interference. The segmented reference plate point clouds were not subjected to any denoising or other post-processing operations, but were directly used for subsequent analysis to ensure that the results reflect the true scanning accuracy.

**Figure 3:**
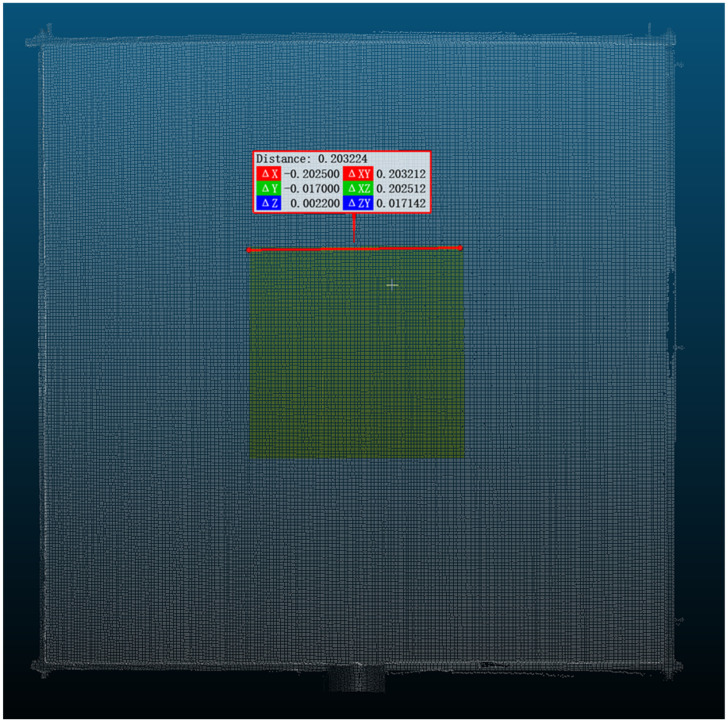
Fitted planar region (yellow area; dimensions: 20 cm × 20 cm).

During the specific data processing procedure, we selected a 20 cm × 20 cm square region centered on each reference plate for plane fitting (as shown in Figure 3). Each selected region contained approximately 20,000 points, providing a sufficient sample size to enhance the reliability of computational results. We employed the least squares method to fit planes and calculated the RMS value of distance residuals between all points in the region and the fitted plane. This approach quantitatively evaluated the fitting accuracy of point cloud data under different incidence angles, effectively revealing how incidence angles affected the measurement precision of point clouds.

The rationale for using raw scanning data without additional denoising treatment lies in the consideration that denoising processes might introduce new errors or alter the distribution characteristics of original data, potentially compromising the authenticity of the experimental results. By directly calculating the RMSs from untreated data, we can more accurately assess the actual measurement precision performance of the laser scanner under various incidence angles.

We experimentally analyzed the variation in point cloud fitting accuracy with an incidence angle for a standard plane under different rotation directions. The experimental results indicate a significant correlation between point cloud fitting accuracy and incidence angle.

**Figure 4:**
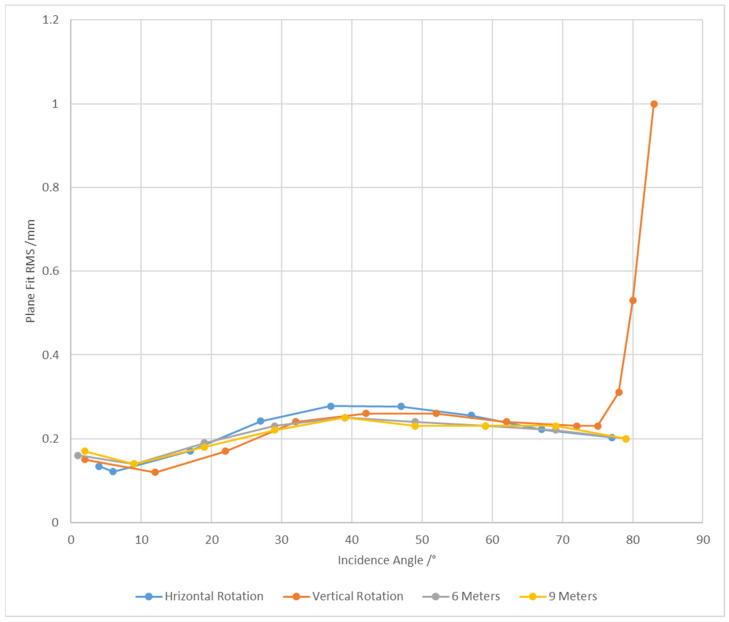
Accuracy under different scanning incident angles and different scanning distances.

In the horizontal rotation experiment (as shown by the blue line in Figure 4), the point cloud fitting residual (RMS) exhibits distinct stage-wise characteristics as the incidence angle varies. When the incidence angle is below 10°, the fitting residual remains stable below 0.15 mm, indicating the high fitting accuracy of the point cloud data. As the incidence angle increases to the range of 10°–40°, the fitting accuracy gradually declines, with the RMS showing an increasing trend. Notably, when the incidence angle exceeds 40°, the fitting accuracy demonstrates a certain degree of recovery, but remains above 0.2 mm overall.

In the vertical rotation experiment (as shown by the orange line in Figure 4), the variation trend of point cloud fitting accuracy exhibits high similarity to that in the horizontal rotation experiment. However, when the incidence angle reaches approximately 80°, the RMS shows a significant increase, indicating more severe degradation in point cloud data accuracy under large incidence angles. Meanwhile, under large incident angles, the accuracy loss caused by vertical angles is greater than that caused by horizontal angles.

Through a comprehensive analysis of the experimental data from both horizontal and vertical rotations, the following conclusions can be drawn:

i When the incident angle is less than 10°, the variation in point cloud fitting accuracy is within 0.05 mm, with a slight upward trend;
ii When the incidence angle is greater than 10° but less than 47°, the point cloud fitting accuracy exhibits a significant declining trend;
iii When the incidence angle exceeds 47°, the fitting accuracy shows slight improvement, although the overall enhancement remains limited.

According to studies by Soudarissanane et al. [25,27], the internally configured angle correction model in laser scanners significantly impacts reflected distance calculations. This model demonstrates more substantial optimization effects under large incidence angles, whereas its effectiveness diminishes with smaller incidence angles. Experimental results indicate that approximately 47° represents the critical point for the instrument’s angle correction model, explaining the phenomenon of gradually improving point cloud accuracy with increasing incidence angles.

In addition, to explore the mechanism by which the point cloud scanning distance affects point cloud quality at the small-scene scale, this study added experimental groups with distances of 6 m and 9 m between the scanner and the flat panel. The experimental procedures strictly followed the processing specifications for horizontal scanning at 3 m that were used previously. The experimental results correspond to the orange line and gray line in Figure 4. Analysis shows that the result data and variation trends under the scanning distances of 6 m and 9 m are basically consistent with those of the 3 m distance group. It can thus be inferred that, within a short distance range (less than 10 m), the impact of changes in scanning distance on point cloud accuracy is negligible.

This experiment not only verifies the point cloud accuracy characteristics of laser scanners under varying incidence angles, but also reveals the influence patterns of their internal angle correction model on measurement results. These findings provide crucial theoretical foundations and practical guidance for optimizing point cloud data processing workflows and enhancing point cloud registration accuracy.

## 3. Principles of Improved ICP Algorithm and Weighted Point Cloud

### 3.1. Principles of Improved ICP Algorithm

Point cloud registration refers to the process of aligning two point clouds through their corresponding point pairs. Given a source point cloud ***P*** and a target point cloud ***Q*** in a 3D space, the objective is to find a rigid transformation ***T****(=**R***, ***t**)* such that the transformed ***P*** optimally aligns with ***Q*** in overlapping regions. Mathematically, this is formulated to minimize the alignment error:

(2) $$E(R,t)=\underset{R,t}{\arg\min}\sum_{(p,q)\in C}{\left\|Rp+t-q\right\|}^{2}$$

where *C* denotes the set of corresponding point pairs, and *p* and *q* are elements in *P* and *Q*, respectively.

Point cloud registration based on corresponding point pairs involves minimizing the objective function in Equation (2). Through iterative optimization, the transformation parameters T that minimize *E(**R***, ***t**)* can be solved. In the classical ICP algorithm, parameters are typically estimated via nonlinear optimization methods, such as SVD decomposition for solving ***T***. This nonlinear approach inherently assumes uniform weighting for all points, failing to incorporate differentiated weighting strategies where individual points contribute variably to the parameter computation. Consequently, the solution may exhibit an inherent bias due to the neglect of point-wise reliability distinctions.

According to the surveying adjustment theory, the objective function (Equation (2)) can be linearized, and the transformation parameters ***T*** can then be solved using the least squares method. The solution process is analogous to the 3D coordinate transformation. During linearization, the Rodrigues rotation matrix principle is adopted to express the rotation matrix ***R*** as a function of three independent parameters. First, a skew-symmetric matrix ***S*** with three independent elements is introduced (Equation (3)). The Rodrigues rotation matrix ***R*** can then be derived from this skew-symmetric matrix via Equation (4):

(3) $$S=\left[\begin{array}{ccc} 0 & -c & -b\\ c & 0 & -a\\ b & a & 0 \end{array}\right]$$

(4) $$R=\left(I+S\right){\left(I-S\right)}^{-1}$$

where ***I*** denotes the third-order identity matrix. Based on the principles of error theory and analysis [28], substituting Equation (3) into Equation (4), followed by substitution into Equation (2) and subsequent linearization, yields the following error equation:

(5) v=Ax+l

where $x={\left[a,b,c,t_{x},t_{y},t_{z}\right]}^{T}$, ***A*** is the coefficient matrix after linearization of the objective function. According to the least squares method, the solution to the above equation is given by the following equation:

(6) $$x={\left(A^{T}PA\right)}^{-1}\left(A^{T}Pl\right)$$

where ***P*** denotes the weight matrix assigned to each corresponding point pair, with the values computed as detailed in Section 3.2. The rigid transformation parameters (***R***, ***t***) can then be solved through iterative refinement.

### 3.2. Incidence Angle-Based Weighting for Point Clouds

#### 3.2.1. Point Cloud Weighting

In general, the quality of point clouds scanned by TLS is poor under large incident angles (exceeding 80°). Experimental results (Section 4.2) further demonstrate that registration accuracy markedly declines when the point clouds involved in registration have incidence angles greater than 85°. To enhance registration accuracy, we assign zero weight to point clouds with incidence angles exceeding 85°, whereas those below 85° are weighted according to a defined weighting function.

Regarding point cloud weighting functions based on scanning incidence angles, two primary methodologies exist: theoretical model-based weighting functions and data-driven weighting functions. As an electro-optical measuring instrument, laser scanners operate under the cosine effect principle [29] inherent to optical measurements. When light rays perpendicularly strike a surface, reflected signal energy concentrates with a higher signal-to-noise ratio. Conversely, when the incident at oblique angles, beam deformation and intensified path scattering occur, leading to increased ranging errors. Consequently, the cosine model serves as the preferred theoretical weighting function, expressed as follows:

(7) $$f(\alpha )=\cos^{k}(\alpha )$$

In the equation, *k* is an empirical parameter. According to the empirical model in Reference [29], when *k* = 1, noise undergoes linear attenuation, making this setting suitable for low-noise environments. Conversely, with *k* = 2/3, the model enhances weights for vertical incidence points while suppressing noise at large angles—an approach widely adopted in LiDAR point cloud processing. Given that this study focuses on point cloud registration rather than noise reduction, the cosine model with *k* = 2/3 is more appropriate as the weighting function.

For data-driven weighting functions, we determine the weighting function by analyzing the pattern of errors varying with incidence angles during standard object scanning. In Section 2 of this paper, the plane fitting accuracy of Terrestrial Laser Scanning (TLS) at different incidence angles has been established, and the detailed data are shown in Table 1. Based on these discrete accuracy observations, we can convert them into weight values through a conversion function. There are two transformation approaches: The first is to directly use the plane fitting accuracy as the precision metric for point clouds at corresponding incidence angles (i.e., mean square error $\sigma_{i}=RMS_{i}$). By designating the RMS value associated with optimal fitting accuracy as the mean square error of unit weight, weights for different incidence angles can be calculated using the weight determination formula (e.g., Equation (8)) from error theory and surveying adjustment theory. The resultant weights are labeled as Weight1 in Table 1. However, since the empirically derived RMS values from plane fitting do not fully represent true point cloud accuracy at given angles—only partially reflecting point cloud quality—the first weighting approach may not represent the most precise solution. Analysis of plane fitting accuracies in Table 1 reveals that the poorest fitting accuracy occurs between 37° and 47° incidence angles. Crucially, point clouds within this angular range constitute a significant portion of the dataset and should not be assigned simply low weights in registration. Therefore, to ensure the appropriate contribution of mid-range incidence angle point clouds, we introduce a second weighting methodology that allocates weights based on relative magnitude relationships between RMS values across incidence angles. This approach assigned the maximum weight value 1 to data with optimal fitting accuracy (RMS = 0.12 mm) and the minimum weight value 0.5 to data with the poorest accuracy (RMS = 0.28 mm). Through the normalization scheme shown in Equation (9), RMS values at different incidence angles are mapped to the weight interval [0.5, 1], yielding the weight distribution results per scanning angle, as shown in Weight2 in Table 1.

(8) $$p_{i}=\frac{{\sigma_{0}}^{2}}{{\sigma_{i}}^{2}}$$

(9) $$p=\frac{RMS_{\max}-x_{rms}}{RMS_{\max}-RMS_{\min}}\times 0.5+0.5$$

**Figure 5:**
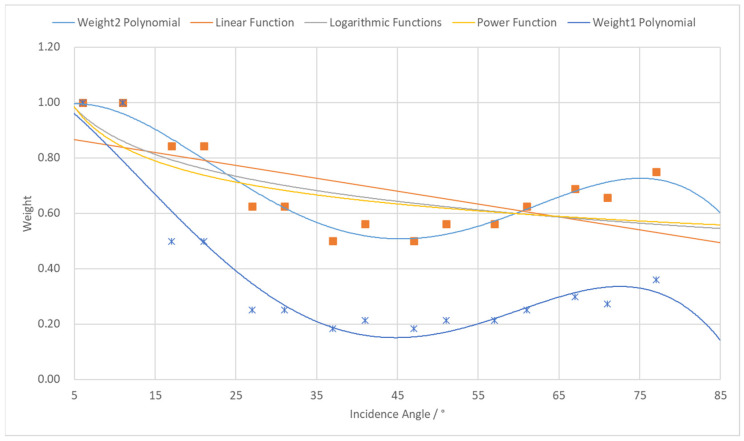
Weight curves under different incidence angles.

Through a curve fitting analysis of the data in Table 1, we aimed to identify the optimal functional relationship describing the variation in point cloud quality under different scanning incidence angles. After testing multiple function forms, the results demonstrate that a fourth-order polynomial function achieves the best fit, with its curve showing high consistency with the actual data distribution (as illustrated in Figure 5). The fourth-order polynomial function is given by Equation (10), where the coefficients derived from the Weight1 weighting scheme are

$$[-1.96085\times 10^{-7},2.87461\times 10^{-5},-9.34778\times 10^{-4},-1.87754\times 10^{-2},1.07302],$$

and those from the Weight2 scheme are

$$[-2.35682\times 10^{-7},3.93177\times 10^{-5},-1.89672\times 10^{-4},1.74796\times 10^{-2},0.942174].$$

(10) $$f(\alpha )=b_{1}\alpha^{4}+b_{2}\alpha^{3}+b_{3}\alpha^{2}+b_{4}\alpha +b_{5}$$

Based on the fundamental principles of laser scanning, there is a negative correlation between the scanning incidence angle and point cloud quality, i.e., the smaller the incidence angle, the higher the point cloud quality. In this experiment, although the overall point cloud quality is higher at smaller incidence angles, there is still some degree of fluctuation in accuracy, which may be attributed to fitting errors inherent in the point cloud data. Given the stability of point cloud quality and its relatively minor variations within the range of small incidence angles, the weights in this interval are uniformly assigned a value of 1.0 in the weighting function design to ensure their dominant role in the registration process.

Based on the above analysis, this study proposes a segmented weighting method applicable to different scanning incidence angles. The specific weighting formula for this method is given in Equation (11).

(11) $$p=\left\{\begin{array}{l} 1,if\;0<\alpha \leq 5^{\circ }\\ f(\alpha ),if\;5^{\circ }<\alpha \leq 85^{\circ }\\ 0,if\;\alpha >85^{\circ } \end{array}\right.$$

where f(α) denotes the weighting function model determined by Equation (7) or (10).

#### 3.2.2. Weighting of Corresponding Point Pairs

The weighted ICP registration algorithm based on incidence angle fundamentally relies on assigning weights to the corresponding point pairs. Since the two point clouds are obtained through independent observation processes, their corresponding point pairs can be treated as mutually independent and uncorrelated observations. From a metrological perspective, according to the error propagation law, the rigorous weight combination formula should be expressed as Equation (12).

(12) $$p=\frac{p_{s}p_{t}}{p_{s}+p_{t}}$$

where $p_{s}$ represents the weight of the source point in the corresponding point pair and $p_{t}$ denotes the weight of the target point in the corresponding point pair.

From a mathematical perspective, the weight of a discrete variable is equivalent to its probability density function. According to probability theory, the joint probability density function of two independent random variables equals the product of their individual probability density functions. By analogy, the combined weight of two independent observations should therefore be the product of their respective weights, as expressed in Equation (13).

(13) $$p=p_{s}p_{t}$$

To validate the compatibility between different weighting functions and weight combination formulae, we designed three weighted algorithms:

i ourWeighted1 incorporates the geodesically derived weighting function (Equation (8) + Equation (10)) and weight combination Equation (12).
ii ourWeighted2 utilizes the analytically derived weighting function (Equation (9) + Equation (10)) with joint weighting (Equation (13)).
iii ourWeighted3 employs the model-driven weighting function (Equation (7)) combined with joint weighting (Equation (13)).

Extensive research indicates that, among various weight combination methods, joint weighting (Equation (13)) demonstrates the most prevalent application. Consequently, while ourWeighted1 uniquely employs the weight combination Equation (12) due to its geodesic theoretical foundation, both ourWeighted2 and ourWeighted3 adopt the joint weighting approach.

### 3.3. Point Cloud Registration Workflow

**Figure 6:**
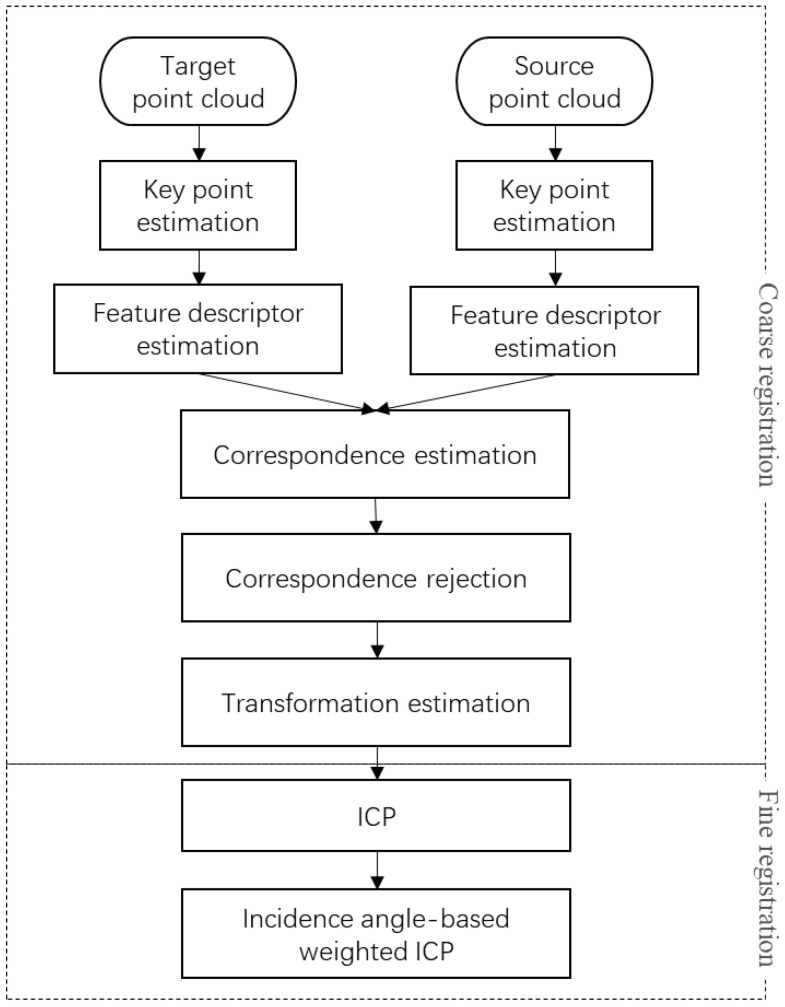
Point cloud registration workflow.

Point cloud registration has evolved into a systematic workflow, typically following the classic coarse-to-fine registration strategy. The improved ICP algorithm proposed by this study still adheres to this general framework, with the complete registration process illustrated in Figure 6. For raw point cloud data acquired by laser scanners, if the resolution is excessively high, downsampling is performed to reduce the computational load; if the resolution is moderate or low, the data are directly sent for subsequent processing.

The coarse registration process primarily consists of the following steps: First, key points are extracted from both the source and target point clouds using algorithms such as Intrinsic Shape Signatures (ISS), Harris3D, or 3D-MSAC. Next, the feature descriptors of these key points are computed, employing methods like Fast Point Feature Histograms (FPFH) or SHOT352. Subsequently, correspondences between the source and target point clouds are established based on their key point descriptors, followed by outlier rejection, to eliminate mismatches. Finally, an initial transformation matrix is computed from the remaining valid correspondences, and the source point cloud undergoes coarse alignment.

After coarse registration, the classic ICP algorithm is applied for fine registration to achieve high-precision alignment. At this stage, the conventional point cloud registration workflow is complete. The weighted registration algorithm proposed by this study, which incorporates scanning incidence angles, serves as a key optimization step built upon the traditional ICP fine registration. Specifically, after fine registration, an additional weighted ICP refinement is performed to further enhance alignment accuracy. The core concept involves assigning different weights to corresponding point pairs based on their scanning incidence angles, computing the Rodrigues transformation matrix via weighted least squares and iteratively updating it until convergence.

**Figure 7:**
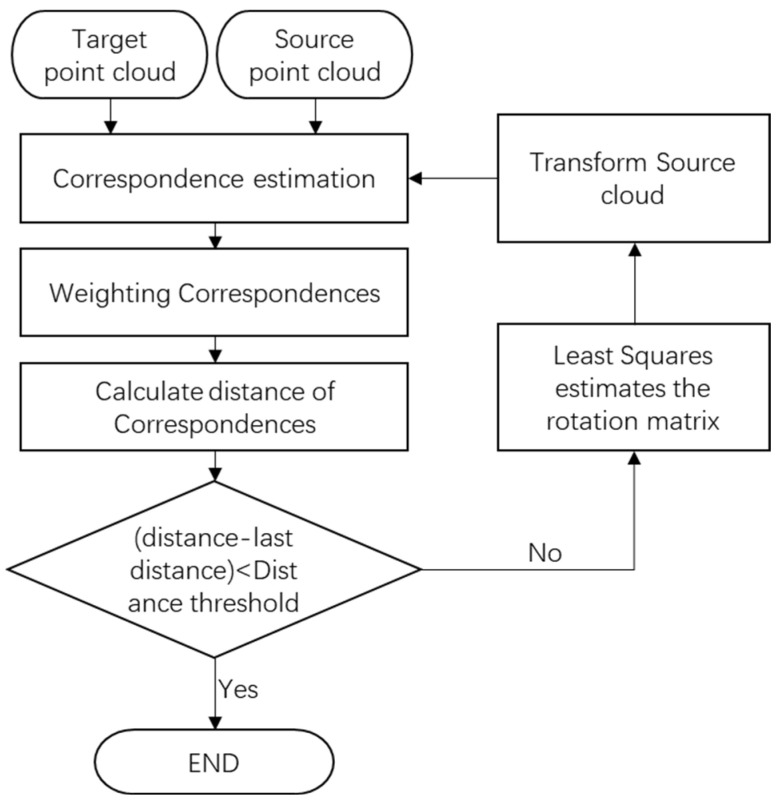
Workflow of the weighted ICP algorithm based on scanning incidence angles.

Figure 7 illustrates the detailed workflow of the incidence angle-based weighted ICP algorithm. The most critical technical aspects lie in the reasonable allocation of weights to the source point cloud and the target point cloud, as well as the synthesis of joint weights for corresponding point pairs. This process not only requires the consideration of the incidence angle characteristics of individual point clouds, but also a comprehensive analysis of the spatial distribution relationship between the two point clouds to ensure the optimality of the final transformation matrix.

In summary, the proposed improved algorithm maintains the original registration framework while introducing an incidence angle-based weighting mechanism to effectively enhance registration accuracy. The key innovations of this method are the angle-dependent weight assignment and the strategy for determining joint weights of corresponding point pairs.

## 4. Experimental Analysis and Precision Evaluation

### 4.1. Evaluation Metrics for Point Cloud Registration Accuracy

In point cloud registration processes, mean square error (MSE) of corresponding point pairs is typically used as a key metric for evaluating registration accuracy. However, this metric may have limitations in practical applications. Specifically, since point cloud data may contain mismatched, corresponding point pairs or noisy point pairs, these factors can significantly affect the MSE-based evaluation results, leading to deviations in accuracy assessment.

To address these issues, this study adopts a more reliable method for evaluating registration accuracy. By uniformly distributing multiple target spheres in the experimental area as external verification conditions, the spatial deviations between the target sphere centers in the registered source point cloud and those in the target point cloud are used for accuracy assessment. Specifically, the three-dimensional coordinate differences in all target sphere centers are calculated, and root mean square error (RMS) is employed as the final accuracy evaluation metric. This geometry-constrained approach not only effectively avoids the influence of mismatched corresponding point pairs and noise, but also more accurately reflects the overall registration accuracy of the point clouds.

**Figure 8:**
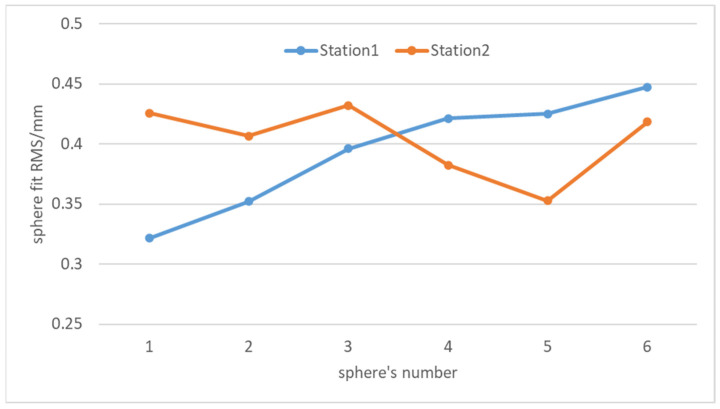
Fitting residuals of target sphere centers.

Figure 8 shows the fitting accuracy of the centers of six target spheres from two scanning stations in the first set of data in Section 4.3. It can be seen in the figure that the absolute fitting accuracy of the target spheres is within 0.5 mm and the maximum deviation of the fitting accuracy between the corresponding target spheres of the two stations is 0.1 mm. The above results indicate that using the position deviation of the target sphere center as an indicator to evaluate registration accuracy is reliable.

### 4.2. Determination of Zero-Weight Incidence Angle Threshold

To evaluate point cloud validity under large incidence angles, this study arranged multiple spherical targets uniformly in a controlled laboratory environment and conducted two scans using a Faro Focus 350 laser scanner to acquire comprehensive scene data containing all targets for subsequent point cloud registration. The experimental procedure consisted of two main steps: First, point clouds with incidence angles exceeding 80° were assigned zero weight to exclude them from the registration process, while all other point clouds were given unit weight 1. The weighted ICP algorithm was then applied for registration. Subsequently, the incidence angle threshold was progressively increased in 1° increments up to 90°, with corresponding registration accuracy being carefully monitored at each increment.

**Figure 9:**
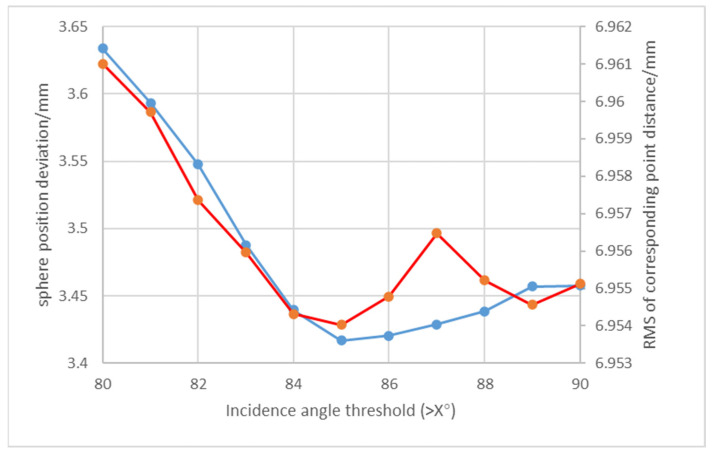
Zero-weight registration fitting plot (Horizontal axis: Incidence angle threshold (>X°). Vertical axis: Registration residual (mm). Red line: RMS of corresponding point pair distances. Blue line: RMS of reference spherical target center positional deviations).

Analysis of the registration results and sphere position deviations in Figure 9 demonstrates that assigning zero weight to point clouds with incidence angles exceeding 85° simultaneously minimizes the RMS of corresponding point pairs and the RMS deviation of sphere center positions. Driven by this empirical evidence, our methodology implements an 85° incidence angle threshold during weighted registration—systematically nullifying weights beyond this critical angle—to ensure optimal alignment accuracy.

### 4.3. Incidence Angle-Weighted Point Cloud Registration

Experimental datasets were acquired using a Faro Focus 350 laser scanner comprising two distinct sets: indoor and outdoor collections. To ensure registration accuracy, standardized spherical targets were deployed as external verification markers in both environments.

**Figure 10:**
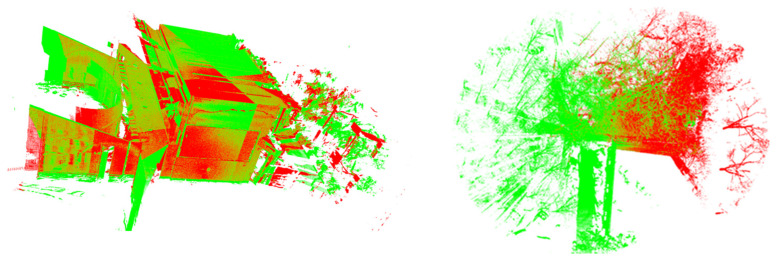
Point cloud diagram after ICP registration. The left side is the indoor scene and the right side is the outdoor scene. Green represents the source point cloud, and red represents the target point cloud.

Following data acquisition, raw point clouds were exported with Faro SCENE software for subsequent processing. This software’s registration capabilities additionally facilitated comparative analysis with alternative methods. We first implemented coarse registration using Fast Point Feature Histogram (FPFH)-based alignment. Subsequently, fine registration was performed using multiple algorithms: conventional ICP, point-to-plane ICP, Generalized ICP (GICP), Go-ICP, Normal-based ICP (NICP), and our proposed weighted ICP approach. Parameters for GICP, Go-ICP, and NICP followed reference implementations from their original publications, while other algorithms used distance constraints equivalent to the average point spacing between correspondences. Visualizations of registered point clouds appear in Figure 10, with quantitative outcomes presented in Table 2. The left side of the figure shows an indoor environment. Within a 10 m range, it is mainly composed of lime walls and floor tiles, which have significant geometric features, making the overall registration process relatively easy. The right side of the figure is an outdoor corner area of a house. Within a 10 m range, the proportion of trees is high, which makes feature extraction more difficult, so the overall registration result is not good.

Table 2 displays the operational results of different fine registration algorithms on two datasets, primarily involving three evaluation metrics: First, RMS (Root Mean Square Error), which represents the error of all corresponding point pair distances under the constraint of one times the average point spacing after registration. Second, the root mean square error of the positional deviations in standard spherical target centers. Third, the computational time required for fine registration. Regarding the first metric, while it can generally reflect the overall accuracy of point cloud registration, due to the fact that the corresponding point pairs are not true conjugate points and inherently contain errors, it shows no significant variation at higher precision levels. In contrast, the second metric—positional deviations of fitted sphere centers—can more accurately reflect precision differences between algorithms.

By comparing registration results across different algorithms, we draw the following conclusions: Although the point-to-plane ICP algorithm exhibits fast operational speed, its registration accuracy is the poorest, indicating that this algorithm achieves higher computational efficiency at the cost of sacrificing some precision. The GICP, Go-ICP, and NICP algorithms show slightly lower accuracy than conventional ICP, but their computational efficiency is the highest, demonstrating significant performance improvements compared to conventional ICP. The commercial software SCENE outperforms conventional ICP and its variant algorithms in terms of sphere positional deviation RMS after registration, while its computational efficiency is only slightly lower than these variants. This suggests that SCENE’s proprietary ICP algorithm has undergone comprehensive optimization and enhancement, improving both registration accuracy and maintaining high operational efficiency. The three weighting algorithms proposed in this study all surpass conventional ICP and its variants in accuracy. Among them, except for the third model-driven weighting algorithm, whose registration accuracy is slightly inferior to SCENE, the other two weighting algorithms exhibit higher registration accuracy than SCENE. Among these three incidence angle-weighted algorithms, the data-driven and empirical model-weighted algorithms demonstrate the highest registration accuracy—approximately 30% higher than conventional ICP and its variants, and about 10% higher than SCENE.

The second dataset contains outdoor scanning data including complex environments such as woods and buildings, resulting in increased noise. Through a comparative analysis of registration results and efficiency across both datasets, we observe that, under complex environmental conditions with high noise levels, all algorithms exhibit varying degrees of reduced registration accuracy, and registration time increases significantly. This indicates that the noise robustness of existing algorithms requires further enhancement. However, under these high-noise and complex environmental conditions, the incidence angle-weighted algorithms proposed in this study still maintain the best registration accuracy.

## 5. Discussion and Conclusions

After years of development, LiDAR point cloud technology has gradually evolved from the early stage of sparse data collection to the current level of refined perception with high density and multiple attributes (such as reflection intensity, semantic information, etc.). In this process, the coordinated development of multiple technologies, such as data compression, data transmission, and environmental perception, has jointly promoted the maturity of the point cloud data processing system [30,31,32]. As a fundamental link in core processes like point cloud stitching and scene reconstruction, the accuracy of point cloud registration directly affects the reliability of subsequent tasks, which constitutes the core motivation for this paper: the optimization of registration methods.

This research systematically investigates the quality characteristics of point cloud data and their intrinsic relationships with scanning incidence angles—a critical issue in point cloud registration. Building upon this foundation, we propose an enhanced ICP registration algorithm incorporating incidence angle weighting, and we innovatively design three distinct weighting fusion methodologies. These contributions establish significant theoretical foundations and practical references for future point cloud registration research.

The results indicate that the three proposed incidence angle-weighted registration algorithms significantly surpass existing algorithms in registration accuracy, with the data-driven incidence angle-weighted algorithm demonstrating particularly outstanding performance—achieving approximately 30% higher accuracy compared to the conventional ICP algorithm and approximately 10% improvement over the commercial SCENE software. However, the current implementation has not yet incorporated optimizations like parallel computing or GPU acceleration, indicating considerable potential for efficiency gains. Future code optimizations are expected to substantially enhance computational performance.

Critically, precise computation of incidence angles constitutes a pivotal determinant of registration accuracy. As defined by the calculation formula in Equation (1), its computational precision is intrinsically dependent on the accuracy of point cloud normal estimation. Consequently, future research should prioritize enhancing normal extraction accuracy in complex environments, thereby elevating the overall efficacy of incidence angle-weighted registration algorithms.

This study focuses on the scan registration challenges specific to TLS systems. Given the device-dependent variations in the relationship between incidence angles and point cloud quality, we recommend device-specific calibration to maximize the advantages of data-driven incidence angle-weighted registration algorithms. Enhanced TLS registration accuracy substantially broadens application potential in industrial automation and precision metrology, particularly demonstrating significant promise for the geometric measurement of large-scale complex structures such as shield tunnel segments and bridge components. These advancements provide critical theoretical foundations and technical support for the wider adoption of static scanning systems.

Beyond the field of industrial measurement, this study has significant limitations in its application scenarios. Take forest environments as an example: the disorderly distribution of trees, sharp topographic fluctuations, and extensive presence of occluders pose immense challenges to point cloud data acquisition and processing, with precise registration standing out as a critical technical bottleneck. The ICP registration algorithm proposed herein, which incorporates weighting based on scanning incident angles, could theoretically optimize the registration process by leveraging the incident angle characteristics of point clouds at different positions within forest scenes. That said, how to accurately capture incident angle data in complex and dynamic forest settings, as well as how to effectively mitigate issues like point cloud data loss and noise interference caused by tree occlusion, still require further in-depth investigation.

In laser scanning applications involving mobile platforms such as airborne and vehicle-mounted systems, the challenges faced by this study’s algorithm are multidimensionally complex. First, during platform operation, dynamic speed variations, persistent vibrations and jolts, and real-time adjustments to travel paths inevitably introduce substantial noise and errors into the point cloud data collected by LiDAR. Second, constrained by the scanning range of LiDAR, frequently shifting environmental scenarios place stringent demands on the algorithm’s real-time performance and computational efficiency. However, the current algorithm—lacking key optimizations such as parallel processing and GPU acceleration—struggles to efficiently handle the massive volumes of point cloud data generated by mobile platforms. Third, non-stationary motion factors like platform shaking and sudden turns can cause random deviations in LiDAR scanning angles, significantly complicating incident angle calculations and thereby severely degrading the performance of the incident angle-weighted registration algorithm. To address these issues, future research must develop systematic optimization strategies to comprehensively enhance the algorithm’s adaptability and processing efficiency in LiDAR applications on mobile platforms.

It is noteworthy that, in the current context of rapid deep learning development, traditional point cloud registration methods are gradually receiving insufficient attention. However, there is limited progress in the research on the influence of point cloud data quality and its generation mechanisms (such as scanning equipment characteristics and incidence angle distribution) on point cloud registration. Future research could further integrate quality assessments of point cloud data with analyses of scanning equipment parameters to establish more comprehensive point cloud processing frameworks, aiming to achieve more robust and efficient scanning measurement applications in complex industrial scenarios.

## Figures and Tables

**Table 1:** Plane fit RMS and weights under different scanning incidence angles.

Angle of Incidence/°	Plane Fit RMS/mm	Weight1	Weight2
4	0.13	0.85	0.97
6	0.12	1.00	1.00
11	0.12	1.00	1.00
17	0.17	0.50	0.50
21	0.17	0.50	0.50
27	0.24	0.25	0.63
31	0.24	0.25	0.63
37	0.28	0.18	0.50
41	0.26	0.21	0.56
47	0.28	0.18	0.50
51	0.26	0.21	0.56
57	0.26	0.21	0.56
61	0.24	0.25	0.63
67	0.22	0.30	0.69
71	0.23	0.27	0.66
77	0.20	0.36	0.75

**Table 2:** Registration accuracy analysis.

Method	Group 1			Group 2		
	RMS/mm	Sphere Center RMS/mm	Time/s	RMS/mm	Sphere Center RMS/mm	Time/s
ICP	21.6	5.2	25	22.5	11.0	100
PlaneICP	23.9	20.5	13	24.3	27.5	19
GICP	21.7	7.5	12	22.7	14.5	18
Go-ICP	21.6	5.5	10	22.6	12.0	17
NICP	21.6	5.3	11	22.6	12.0	18
SCENE	----	3.9	17	----	8.0	23
ourWeighted1	21.5	3.5	17	22.4	7.5	100
ourWeighted2	21.5	3.2	30	22.4	7.1	112
ourWeighted3	21.5	4.1	18	22.4	8.2	108

---- indicates no data available for this item.

## Data Availability

All data involved in this paper were collected on-site using a Faro Focus350 laser scanner, including the scanning measurement data of standard steel plates at distances of 3 m, 6 m, and 9 m, as well as indoor and outdoor registration data. Given that the volume of the original measured data is relatively large (exceeding 10G), it is inconvenient to upload directly. If needed, you can contact the author via email, and we will transmit the data through cloud storage. The author promises that the provided measured data are true and valid.
